# Circulating mediators of remote ischemic preconditioning: search for the missing link between non-lethal ischemia and cardioprotection

**DOI:** 10.18632/oncotarget.26537

**Published:** 2019-01-04

**Authors:** Muntasir Billah, Anisyah Ridiandries, Usaid Allahwala, Harshini Mudaliar, Anthony Dona, Stephen Hunyor, Levon M. Khachigian, Ravinay Bhindi

**Affiliations:** ^1^ Department of Cardiology, Kolling Institute, Northern Sydney Local Health District, St Leonards, NSW, Australia; ^2^ Sydney Medical School Northern, University of Sydney, Sydney, NSW, Australia; ^3^ Vascular Biology and Translational Research, School of Medical Sciences, University of New South Wales, Sydney, NSW, Australia

**Keywords:** remote preconditioning, cardioprotection, myocardial infarction, ischemia-reperfusion, circulating mediators

## Abstract

Acute myocardial infarction (AMI) is one of the leading causes of mortality and morbidity worldwide. There has been an extensive search for cardioprotective therapies to reduce myocardial ischemia-reperfusion (I/R) injury. Remote ischemic preconditioning (RIPC) is a phenomenon that relies on the body's endogenous protective modalities against I/R injury. In RIPC, non-lethal brief I/R of one organ or tissue confers protection against subsequent lethal I/R injury in an organ remote to the briefly ischemic organ or tissue. Initially it was believed to be limited to direct myocardial protection, however it soon became apparent that RIPC applied to other organs such as kidney, liver, intestine, skeletal muscle can reduce myocardial infarct size. Intriguing discoveries have been made in extending the concept of RIPC to other organs than the heart. Over the years, the underlying mechanisms of RIPC have been widely sought and discussed. The involvement of blood-borne factors as mediators of RIPC has been suggested by a number of research groups. The main purpose of this review article is to summarize the possible circulating mediators of RIPC, and recent studies to establish the clinical efficacy of these mediators in cardioprotection from lethal I/R injury.

## INTRODUCTION

Impaired coronary circulation during myocardial ischemia leads to lethal hypoxic injury. Rapid restoration of coronary blood flow through either thrombolytic therapy or percutaneous coronary intervention (PCI) is essential to limiting myocardial infarction (MI) size, preserving left ventricular (LV) ejection fraction and preventing LV remodeling. Revascularization via either thrombolytic therapy or PCI reduces the mortality rate of patients suffering a heart attack by almost 50% [1]. Paradoxically, restoration of oxygenated blood to the ischemic heart after revascularization aggravates tissue damage, a phenomenon known as ischemia reperfusion (I/R) injury [2] considered to be mediated by calcium overload, oxidative stress and inflammation [3]. This prompted a search for novel therapeutic strategies to protect the heart against I/R injury to improve clinical outcomes of the patients presenting with MI.

The current therapeutic strategy to I/R injury is ischemic conditioning, which is an endogenous approach to protect the heart against acute I/R injury. Murry *et al.* used a canine model to provide the first demonstration that brief episodes of non-lethal I/R to the heart prior to sustained ischemia can dramatically reduce MI size, an endogenous cardioprotective phenomenon termed ischemic preconditioning (IPC) [4]. IPC has been well studied and found to reduce I/R associated damage to other organs including the lung [5], kidney [6], liver [7], skeletal muscle [8], intestine [9], brain [10] and improve post-operative recovery from cardiac surgeries [11]. The potential clinical application of IPC is restricted to elective cardiac surgeries, where the timing of ischemic insult is well controlled. However, patients with acute myocardial infarction (AMI) presented with blocked coronary arteries, making it impossible to precondition the heart. Ischemic postconditioning (IPost), where a non-lethal I/R is performed to the heart by interrupting the PCI-induced reperfusion, delivers a similar outcome to IPC making it a better strategy to treat patients with AMI. Both IPC and IPost require interventional approaches, which limit application in clinical settings. In contrast to directly preconditioning the target organ, Przyklenk and Whittaker in 1993 made the intriguing discovery that preconditioning the heart does not limit its efficacy to the perfused area of the coronary artery, but was extended to remote myocardial tissue [12]. Similarly, Liauw *et al.* showed that skeletal muscle can be protected against I/R by preconditioning the contralateral skeletal muscle [13]. This discovery facilitated the extension of preconditioning techniques to protect other organs beyond the heart. This approach of remotely protecting a target organ through ischemic preconditioning is known as remote ischemic preconditioning (RIPC). A major advance in myocardial RIPC came with the use of skeletal muscle as the origin of RIPC stimulus and brief I/R produced with a tourniquet applied to one of the hind limbs of pig [14]. This lead to a blood pressure measuring cuff around the arm to achieve the RIPC stimulus making it possible to accommodate most of the clinical settings of acute I/R injury. In a non-invasive approach, RIPC has the capacity to protect the organ or tissue whether applied prior to I/R (RIPC), after ischemia but prior to reperfusion (PerC) [15] or during reperfusion (remote ischemic postconditioning, RIPost) [16]. Pryds and colleagues demonstrated the long term effect of RIPC on heart failure patients and reported that though RIPC does not improve left ventricular ejection fraction (LVEF) but reduces blood pressure and NT-proBNP in patients with compensated chronic ischemic heart failure [17] and may reduce the risk of thrombosis by stimulating fibrinolysis [18]. Table 1 summarizes the key clinical trials on the effect of RIPC prior to coronary artery bypass graft (CABG) and PCI. Previous review papers by Hausenloy and Yellon in 2008 [19] and Costa *et al.* in 2013 [20] discussed the cardioprotective pathways induced by RIPC. The present review focuses on the circulating mediators of RIPC that underpin signal transduction mechanisms from the remote organ to the target organ.

**Table 1:** Key clinical trials of RIPC

First author	Nature of trial	Number of participants analyzed (RIPC / Control)	RIPC protocol	Cardioprotection
**Coronary artery bypass graft**				
Hong et al. [218]	RCT	35/35	4 cycles of 5 min I/R on lower limb	Yes
Lucchinetti et al. [214]	RCT	27/28	4 cycles of 5 min I/R of leg	No
Hausenloy et al. [219]	RCT	27:30	3 cycles of 5 min I/R of right upper limb	Yes
Candilio et al. [220]	RCT	89/89	2 cycles of simultaneous 5 min I/R on upper arm and upper thigh	Yes
Venugopal et al. [221]	RCT	23/22	3 cycles of 5 min I/R of right forearm	Yes
Hausenloy et al. [215]	Multicenter RCT	801/811	4 cycles of 5 min I/R of upper arm	No
Krogstad et al. [216]	RCT	45/47	3 cycles of 5 min I/R of upper arm	No
Hong et al. [222]	RCT	644/636	4 cycles of 5 min I/R of upper limb as RIPC and 4 cycles of 5 min I/R of upper limb as RIPost	No
Meybohm et al. [207]	Multicenter RCT	692/693	4 cycles of 5 min I/R of upper arm	No
**Percutaneous coronary intervention**				
Pryds et al.[223]	Post-hoc analysis of RCT	166:167	4 cycles of 5 min I/R of upper arm	Yes
Sloth et al. [224]	Post-hoc analysis of RCT	71:68	4 cycles of 5 min I/R of upper arm	Yes
Pryds et al. [225]	Post-hoc analysis of RCT	71:68	4 cycles of 5 min I/R of upper arm	Yes
Botker et al. [226]	RCT	126: 125	4 cycles of 5 min I/R of upper arm	Yes
Prasad et al. [217]	RCT	47:48	3 cycles of 3 min I/R of upper arm	No
Verouhis et al. [199].	RCT	60:55	1 cycle of 5 min I/R of left thigh before PCI and 4 cycles of 5 min I/R of left thigh post reperfusion	Neutral

Note: RCT: Randomized control trial, I/R: Ischemia-reperfusion.

## INTER-ORGAN PRECONDITIONING

The effect of RIPC is not confined to one organ but impacts multiple organs. Similarly, different organs can be used as the RIPC site. Table 2 summarizes the key findings on inter-organ preconditioning studies. Briefly, applying RIPC stimulus to different organs has been shown to protect various target organs from I/R injury. These protective effects include reduced infarct size, decrease arrhythmia, improved lung and liver function (Table 1).

**Table 2:** Key studies on inter-organ preconditioning

Study (RIPC Site)	Species	Target organ	Result
**Renal**			
McClanahan *et al.* [227]	Rabbit	Heart	↓Infarct size
Gho *et al.* [22]	Rat	Heart	↓Infarct size
Verdouw *et al.* [228]	Pig	Heart	↓Infarct size
Pell *et al.* [229]	Rabbit	Heart	↓Infarct size
Takaoka *et al.*[52]	Rabbit	Heart	↓Infarct size and improved myocardial energy metabolism
Diwan *et al.* [230]	Rat	Heart	Conferred cardioprotection by NFkB activation followed by opening of K(ATP) channels
Lang *et al.* [32]	Rat	Heart	↓Infarct size
Singh *et al.* [231]	Rat	Heart	↓Infarct size and proposed the involvement of angiotensin AT(1) receptors in renal preconditioning
Kant *et al.* [232]	Rat	Heart	Reduced myocardial injury through inhibition of hypoxia inducible factor-prolyl 4-hydroxylases
**Small Intestine**			
Gho *et al.* [22]	Rat	Heart	↓Infarct size
Verdouw *et al.* [228]	Pig	Heart	↓Infarct size
Patel *et al.* [233]	Rat	Heart	↓Infarct size
Heidbreder *et al.*[234]	Rat	Heart	↓Infarct size and activated p38 MAPK, ERK ½ and JNK ½ selectively in the intestine but not in the heart
**Liver**			
Ates *et al.* [235]	Rat	Kidney	Improved creatine clearance and improvement in hepatic histopathologic parameters
Brzozowski *et al.* [236]	Rat	Gut	Reduced gastric mucosa lesion
**Brain**			
Tapuria *et al.* [237]	Rat	Liver	Improved hepatic microcirculation and reduced hepatic I/R injury.
**Hind Limb**			
Oxman *et al.* [238]	Rat	Heart	Decreased arrhythmias
Birnbaum *et al.*[239]	Rabbit	Heart	Reduced MI size
Liauw *et al.* [13]	Rat	Thigh muscle	Reduced muscle necrosis
Kharbanda *et al.*[14]	Pig	Heart	Reduced MI size
Gunaydin *et al.* [240]	Human	Heart	Enhanced anaerobic glycolysis to protect heart
Xia *et al.* [241]	Sheep	Lung	Protected lung from repeated coronary artery occlusion (CAO) and reperfusion mimicking multi-vessel off-pump coronary artery bypass (OPCAB) revascularization and decreased pulmonary vascular resistance
Addison *et al.* [242]	Pig	Skeletal muscle	Protected global skeletal muscle against infarction
Kuntscher *et al.* [243]	Rat	Adipocutaneous flaps	Decreased flap necrosis
Kuntscher *et al.* [244]	Rat	Cremasteric muscle flaps	Decreased flap necrosis
Kuntscher *et al.* [245]	Rat	Epigastric adipocutaneous flaps	Decreased flap necrosis
Moses *et al.* [246]	Pig	Latissimus dorsi (LD) muscle flaps	Decreased flap infarction
Wang *et al.* [247]	Rat	Cremaster flap	Decreased flap necrosis
Harkin *et al.* [248]	Pig	Lung	Reduced acute remote lung damage against systemic inflammatory response from limb I/R injury
Li *et al.* [249]	Mice	Heart	Protected LV function and reduced infarction size
Konstantinov *et al.* [24]	Pig	Heart	Reduced I/R injury in the brain-dead donor heart following orthotopic heart transplantation.
Chen *et al.* [250]	Rat	Heart	Reduced infarction size
Chen *et al.* [251]	Rat	Heart	Reduced infarction size through free radical pathway
Luokogeorgakis *et al.* [252]	Human	Forearm	Preserved endothelial function in the forearm
Waldow *et al.* [253]	Pig	Lung	Protected lung function and reduced the plasma interleukin-1beta level
Kristiansen *et al.* [254]	Rat	Heart	Reduced MI size through a mechanism involving mitochondrial K(ATP) channels and improved LV function during reperfusion
Zhang *et al.* [255]	Rat	Heart	Reduced infarction size and I/R-induced plasma lactate dehydrogenase level
Dave *et al.* [256]	Rat	Heart	Increased neuroprotection from asphyxial cardiac arrest
Kanoria *et al.* [257]	Rabbit	Liver	Reduced liver I/R injury and improved liver function
Lai *et al.* [258]	Rat	Liver	RIPC stimulated heme oxygenase-1 expression in liver tissue and associated with liver protection from I/R injury
Cheung *et al.* [259]	Human	Heart	Postoperative improvement in lung function and reduction in plasma troponin-I level
Mudaliar *et al*. [260]	Rat	Heart	↓ Infarct size through JAK-STAT pathway upregulation

Note: CAO: coronary artery occlusion, OCABG: off-pump coronary artery bypass, LV: left ventricular

## MECHANISMS UNDERLYING RIPC

**Figure 1:**
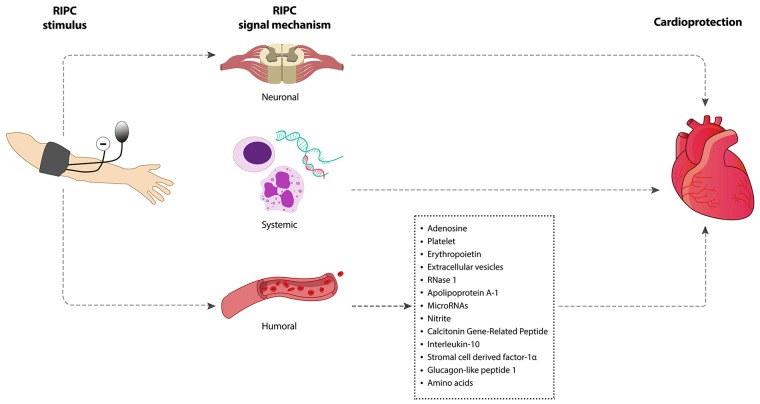
Signaling mechanisms underpinning RIPC-induced cardioprotection Intermittent limb ischemia and reperfusion confers cardioprotection through neuronal, systemic and humoral mechanism.

The underlying mechanisms through which brief episodes of I/R in an organ or tissue transduces a protective signal to a distant organ and renders it resistant to sustained I/R injury is not fully understood. Some studies suggest there is similarity in the mechanistic process of direct preconditioning and RIPC. Based on current knowledge, this can be divided into three major parts: (i) the humoral (ii) the neuronal pathway, and (iii) the systemic pathway (Figure 1). However, whether these pathways independently exert protective effect on the target organ or that crosstalk is involved is not well understood. Table 3 summarizes the major animal studies on the mechanisms of RIPC-induced protection of target organs. The studies focused on the neural, humoral and systemic pathways of RIPC. Apart from Jones *et al.* [21] these studies applied intermittent I/R as RIPC stimulus. More information may be sourced from the references provided.

**Table 3:** Key animal studies on mechanisms of RIPC-induced organ protection

Study	Model	RIPC Stimulus	Target organ	Effect on the target organ	Mechanistic Insight	Comments
Gho *et al.* [22]	Rats	i) 15 min CAO 10 min reperfusion ii) 15 min MAO 10 min reperfusion iii) 15 min renal artery occlusion 10 min reperfusion	Heart	↓Infarct size	Neural pathway	Ganglion blocker abolished RIPC effect while maintaining direct preconditioning effect
Schoem-aker and Heijningen [92]	Rats	15 min intestinal ischemia 10 min reperfusion	Heart	↓Infarct size	Neural pathway	Bradykinin, Hexamethonium may be activating the neural pathway
Liem *et al.* [37]	Rats	15 min intestinal ischemia 10 min reperfusion	Heart	↓Infarct size	Neural pathway	RIPC mediated adenosine upregulation conferred protection against I/R and adenosine receptor blocking abolished RIPC protection
Dong *et al.* [38]	Rats	10 min hind limb ischemia 10 min reperfusion	Heart	↓Infarct size	Neural pathway	Dissecting femoral nerve of the hind limb prior to RIPC abolished the RIPC effect on the target organ
Tang *et al.* [100]	Rabbits	10 min intestinal ischemia 15mins reperfusion	Heart	↓Infarct size	Neural Pathway	RIPC mediated protective effect is associated with capsaicin-sensitive sensory nerves activation
Brzozowski *et al.* [236]	Rats	i) 2 cycles of 5 min left anterior descending artery occlusion and 5 min reperfusion ii) 2 cycles of 5 min occlusion of common hepatic artery and portal vein followed by 10 min reperfusion	Gut	↓Gastric mucosal lesion	Neural pathway	Conferred gastroprotection via vagal and sensory nerve mediated vasodilatory mediators
Weinbrenner *et al.* [23]	Rabbits	15 min infra-renal aortic ischemia 10 min reperfusion	Heart	↓Infarct size	Humoral Pathway	Neural ganglion blocker could not abolish RIPC effect
Patel *et al.* [233]	Rats	15 min MAO followed by 10 min reperfusion	Heart	↓Infarct size	Neural pathway	Preconditioning-induced opioid release and opioid receptor activation protected the myocardium from ischemic injury. Opioid receptor antagonist abolished the protection
Konstantinov *et al.* [41]	Human	3 cycles of 5 min forearm ischemia and 5 min reperfusion	Leukocytes	Decreased CD11B expression on leukocytes	Systemic pathway	Suppressed pro-inflammatory and pro-apoptotic gene transcription
Wolfrum *et al.* [105]	Rats	15min MAO 15min reperfusion	Heart	↓Infarct size	Humoral & Neural pathway	RIPC increased plasma CGRP level and CGRP activated PKCε via neural pathway
Zhang *et al.* [255]	Rats	3 cycles of 5 min femoral artery occlusion followed by 5 min reperfusion	Heart	↓Infarct size	Neural pathway	Activation of kappa-opioid receptors provided cardioprotection induced by RIPC and mPTP inhibition is downstream of kappa-opioid receptor activation
Konstantinov *et al.* [24]	Pigs	4 cycles of 5 min lower limb ischemia 5 min reperfusion	Heart	↓Infarct size	Humoral pathway	K ATP channel dependent mechanism provided RIPC-induced cardioprotection and excluded afferent neurogenic mechanism
Jones *et al.* [21]	Mice	Abdominal incision for RIPC of trauma	Heart	↓Infarct size	Neural pathway	Skin nociception provided cardioprotection through neurogenic signaling involving spinal cords and activation of cardiac sensory and sympathetic nerves

Note: CAO: coronary artery occlusion, MAO: mesenteric artery occlusion.

### Humoral pathway

Multiple studies support the theory of blood borne mediators as a signal transduction mechanism and the requirement for a period of reperfusion to washout humoral factors generated by RIPC [22], [23]. These protective substances circulate via the bloodstream and upon reaching the target organ bind to respective receptors and activate intracellular signaling pathways. Humoral pathway involvement in RIPC was demonstrated by Konstantinov and colleagues [24]. Denervated donor heart recipient pigs that underwent remote limb preconditioning showed significant reduction of MI size, which provides evidence for the concept of humoral-mediated cardioprotection by RIPC. Dickson and colleagues showed for the first time that RIPC could elicit cross species protection [25–27]. These studies explored transfusing blood from preconditioned rabbit hearts and kidneys to a non-preconditioned isolated rabbit heart and showed recovery of the heart from myocardial I/R injury by reducing the infarct size. These authors also showed that coronary effluent from a preconditioned *ex-vivo* rabbit heart could potentiate the similar infarction limiting effect and improve left ventricle function [28]. Shimizu *et al.* reported similar cross species protection after using plasma dialysate from remote preconditioned rabbit and human blood to protect *ex vivo* rabbit heart from I/R injury [29]. These authors also confirmed that the transferrable factors are hydrophobic in nature and <15 kDa in size. Serejo *et al.* provided evidence that the humoral factors released from the ischemic preconditioned heart were thermolabile, hydrophobic, >3.5 kDa and conferred cardioprotection via the activation of protein kinase C (PKC) [30]. Breivik *et al.* also reported the presence of <30 kDa hydrophobic factors in the coronary IPC effluent, which can confer cardioprotection via the PI3K/AKT pathway [31]. Interestingly, proteomic analysis of renal RIPC conducted by Lang and colleagues could not detect any cytoprotective factors larger than 8 kDa [32]. The humoral factors responsible for the RIPC effect on the target organs still remain unclear and investigation into the factors responsible continues. Identifying the potential humoral mediators of RIPC could assist in confirming that the threshold for a RIPC response has been achieved [33].

### Neural pathway

A neural pathway is one that connects one part of the nervous system with another by way of axons. Evidence suggests that intact neural pathway is essential for the remote organ or tissue to convey protective signal to the target organ during the process of RIPC. Denervation of the neural pathway in the remote organ abolishes RIPC protection [34, 35]. In contrast Konstantinov and colleagues show that denervation of the recipient donor heart does not eliminate the RIPC-induced MI size reduction effect [24]. However, the exact role of the afferent and efferent component of the neural pathway is unclear. The involvement of the neural pathway in RIPC-mediated cardioprotection was explored by Gho *et al.* who demonstrated that transient occlusion of the anterior mesenteric artery can mediate cardioprotection, which can be abrogated by ganglionic blockers [22]. This finding was supported with the proposition that RIPC propels the production of autacoids such as adenosine, bradykinin, CGRP in the remote preconditioned organ, which stimulates afferent nerves and relays the neural signal to the myocardium via the efferent nerve fibers. Furthermore, Ding *et al.* explored the role of renal nerve-mediated cardioprotection [36]. They confirmed that renal nerve resection abolished renal preconditioning-induced cardioprotection. Liem *et al.* provided confirmatory evidence implicating adenosine in a neural pathway of cardioprotection [37]. They reported that adenosine released by the mesenteric artery during preconditioning reduced myocardial infarct size from 68% to 48%, a protective effect that was reversed by the ganglionic blocker hexamethonium. In addition, intramesenteric artery infusion of adenosine mimicked similar cardioprotection as mesenteric artery-induced preconditioning, which could be abolished by hexamethonium. From these findings, the investigators concluded that locally released adenosine during mesenteric artery preconditioning stimulates afferent nerves in the mesenteric bed which helps activate myocardial adenosine receptors. Dong *et al.* demonstrated that dissecting the femoral nerve prior RIPC does not protect the myocardium against I/R injury and suggested that an intact neural pathway was required for the sensory afferent neural signaling from the preconditioned limb [38]. A study carried out by Jones and colleagues showed that instead of IPC, abdominal slit in mice activates the cardiac sensory and sympathetic nerves. This procedure elicits cardioprotection via bradykinin (a known hormone and neurotransmitter) release in the heart by the sympathetic nerves and bradykinin dependent activation of PKC-ε [21].

### Systemic pathway

Remote ischemic conditioning has been shown to provoke a systemic response by modulating inflammatory cells either post-transcriptionally or through transcriptional regulation [39]. In contrast to the humoral pathway, the systemic pathway involves the inflammatory cells and provokes an inflammatory response to confer the RIPC signal. Kharbanda *et al.* previously showed that RIPC reduced expression of neutrophil CD11b and platelet-neutrophil complexes in humans [40]. In 2004, Konstantinov *et al.* used microarray analysis of blood samples from healthy human volunteers subjected to forearm preconditioning to reveal that preconditioning suppressed genes regulating cytokine production, leukocyte chemotaxis, adhesion and migration, exocytosis, innate immunity, signaling pathways, and apoptosis, while up-regulating anti-inflammatory genes such as HSP-70 and calpastatin [41]. Later, the same group provided evidence to show that RIPC upregulated genes associated with growth and metabolism, DNA repair and redox regulation. IPC attenuated P-selectin expression in liver and prevented neutrophil infiltration in lung, stomach, pancreas, small intestine and colon via inhibition of systemic TNF-α production [42]. In another study, Albrecht *et al.* reported similar findings in human, showing that within the early phase of RIPC, serum cytokines were upregulated [43]. It may be that, cytokines function as both pro- and anti-inflammatory mediators in ischemic conditioning to prepare the target organ to mitigate the tissue damage. This group's findings showed concurrent increase of IL-8, IL-1β, TNF-α and concurrent cardioprotection due to increased neutrophil infiltration after right atrial bypass surgery [43].

## CIRCULATING MEDIATORS OF ISCHEMIC CONDITIONING

Remote preconditioned organ may require a ‘flush’ after brief ischemia in order to transport humoral factors to the target organ generated by the preconditioning stimulus [19]. Number of studies focused on identifying the nature of the circulating mediators in bloodstream, which likely carries the preconditioning signal from the remote organ to the target organ [29, 30, 32]. Though the actual identity remains elusive, below we discuss a number of blood borne mediators known to be involved in mediating the RIPC-induced protection to the heart against I/R injury.

### Adenosine

Adenosine plays an important role in many biochemical processes including energy transfer, signal transduction or as a neuromodulator. Myocardium expresses several adenosine receptors [44]. Activation of adenosine receptors provides a myocardial preconditioning like affect in rabbits [45], dogs [46] and pigs [47]. Adenosine activates toxin sensitive G protein, which can activate ATP sensitive K^+^ channel [48]. Surendra and colleagues previously showed that δ-opioid and κ-opioid receptors interact with adenosine A1 receptors to mediate RIPC-induced cardioprotection [49]. Furthermore, activation of the ATP sensitive K^+^ channel has been demonstrated to be involved in myocardial preconditioning in dogs. Conversely, neither adenosine [50] nor the ATP sensitive K^+^ channel [51] confer a protective effect in myocardial preconditioning in a similarly designed rat model. However, using the adenosine receptor inhibitor, Takaoka and colleagues have shown the contribution of adenosine in RIPC through renal artery I/R in a rabbit model and reported reduction of MI size [52]. AMISTAD-I trial with `reduced MI size by 50% [53]. This study was followed by a large-scale AMISTAD II trial which recruited over 2000 patients undergoing PCI or thrombolysis for anterior STEMI but found no effect of adenosine infusion in terms of death or heart failure [54, 55]. However, patients who received PCI within the first few hours (<3.17 h) from the onset of the symptoms and received adenosine infusion showed lower mortality and heart failure in *post hoc* analysis.

### Platelet dependent signaling

Platelet activation by mechanical pressure, ischemia or tissue damage invokes a systemic response as one of the first lines of protective defense. Oberkofler *et al.* addressed the role of activated platelet derived serotonin in RIPC-mediated liver and kidney protection against I/R [56]. This group showed that RIPC-induced reduction of I/R injury is associated with prompt release of serotonin by activated platelets and serotonin-mediated activation of the VEGF/IL-10/MMP8 pathway. Reducing platelet numbers by more than 90% by inducing thrombocytopenia with an anti-CD41 injection abolished the RIPC-mediated protection against liver I/R injury and increased of both ALT/AST levels. RIPC displayed a 40% reduction in platelet numbers and when platelet activation was blocked with the platelet-activating factor (PAF) receptor antagonist SM-12502, the effect of RIPC was eliminated. This supports the importance of platelet activation for RIPC. This study demonstrated that serotonin plays a crucial role in platelet dependent protection mechanisms. When tryptophan hydroxylase 1 (Tph1^-/-^) mice lacking platelet serotonin were subjected to RIPC prior to hepatic I/R injury, they did not show any reduction in I/R injury. This result however was reversed by injecting 5HTP, a serotonin precursor. Further studies aimed at elucidating the downstream effector of serotonin found that RIPC resulted in significant increase in serum vascular endothelial growth factor (VEGF) levels, which is cardioprotective [57] and can be upregulated by serotonin [58]. Recombinant VEGF-A treatment prior to I/R resulted in similar effects of RIPC, whilst anti-VEGF antibody treatment abolished the RIPC effect, supporting the importance of circulating VEGF in RIPC. In addition, RIPC upregulated IL-10 and MMP-8 expression in liver, heart, kidney, lung, and intestine. Similar results have been achieved by treating mice with serotonin. Despite these results, IL-10^-/-^ mice with MMP-8 inhibition showed total amelioration of the RIPC-mediated protection, implicating the involvement of serotonin-VEGF-IL-10/MMP-8 axis in facilitating the systemic organ protection induced by platelet activation.

### Erythropoietin

Erythropoietin (EPO) is a circulatory 30 kDa glycoprotein, a principal regulator of red blood cell production. This 165-amino acid long protein has 4 glycosylated chains, which renders its biological activity and protects it from oxygen radicals. Previous studies have shown that exogenous administration of EPO as well as increased endogenous serum EPO improves MI pathobiology by reducing the infarction size as well as improving cardiac function in a time dependent manner [59–62]. EPO can prevent apoptotic cell death in isolated cardiomyocytes by activating the JAK2-PI3K-AKT signaling as well as by suppressing glycogen synthase kinase-3β, a serine-threonine kinase that induces apoptosis in various cell types including vascular smooth muscle cells, cardiomyocytes, and neurons [63–68]. Previously Oba *et al.* demonstrated that renal nerve-mediated EPO release is essential for RIPC-induced cardioprotection [69]. In a mouse model with 4 cycles of 5 min hypoxia and reoxygenation there was an increase in serum EPO levels in a time dependent manner, which peaked 1 h post RIPC and returned to basal levels 24 h post RIPC. Similar findings were obtained in a human study of RIPC with 3 cycles of 5 min of upper arm ischemia and 5 min reperfusion. In both cases, cardioprotective cytokines such as interleukin-6, G-CSF, and leukemia inhibitory factor in serum did not change. RIPC reduced renal blood flow (RBF) in mice with production and secretion of EPO, which is regulated by tissue oxygen supply. Hypoxic tissue results in increased production of EPO and EPO receptors (EPOR). RIPC activated the HIF-1α-EPO pathway and reduced MI size after 2 h of permanent occlusion of the left coronary artery. In addition, injecting the anti-EPO neutralizing antibody 24 h prior to RIPC abolished the RIPC-induced attenuation of infarct size. However, there is controversy regarding the role of renal nerves in EPO production. Oba's group also found that renal denervation abolished RIPC-mediated serum EPO elevation and hence the reduction of MI size. This study elegantly demonstrated that RIPC-mediated EPO secretion by the kidney is renal nerve activity-dependent and can limit the MI pathology. RIPC failed to confer cardioprotection in rats with renal failure, which may be attributed to the lack of EPO release during renal failure [70]. Further insight into signals transduced from the preconditioned limb to the kidney to stimulate the process of EPO production is still needed.

### Extracellular vesicles

Extracellular vesicles (EV) is a collective term for vesicles which are 30–100 nm (exosomes) and 100–1000 nm (microvesicles/micropartciles) in diameter. EV's have many functions and abilities, one of which is to act as transport for proteins, miRNAs and signaling between cells [71]. Giricz *et al.* demonstrated in the Langerdorff rat heart model that preconditioning increases the number of extracellular vesicles (EV) released into the coronary perfusate [72]. They found that perfusate collected from preconditioned heart reduced MI size significantly, whereas perfusate depleted of EV showed no signs of reducing the infarction size. A subsequent study by Vincencio *et al.* determined that endogenous plasma exosomes from rats and human subjected to remote ischemic preconditioning activated pro-survival signally in cardiomyocytes through toll-like receptor 4, leading to activation of ERK1/2, MAPK signaling pathways leading to phosphorylation of the cardioprotective heat shock protein 27 (HSP27) [73]. Similarly, Davidson *et al.* demonstrated that pre-incubation of purified HUVEC exosomes reduced the cell death of rat cardiomyocytes after stimulation of ischemic reperfusion injury [74]. In this case, exosomes activated the ERK1/2, MAPK signaling pathway which contributes to cardioprotection. Furthermore, Mighua *et al*. found increased levels of miR-24 in plasma exosomes of rats subjected to RIPC [75]. They found that miR-24 production after RIPC was able to reduced cardiomyocyte apoptosis, reduce infarct size and improve heart function. Whilst these studies are promising there is still more research to be conducted to understand the signaling and effector mechanism of the extracellular vesicles.

### RNase1

Extracellular RNA (eRNA) functions as an early signal for tissue stress or damage and provides a signal to release cytokines like TNF-α and mediate leukocyte trafficking and consequently inflammatory processes [76]. RNase1 has been shown to prevent the crosstalk between eRNA and TNF-α in mouse cardiac I/R injury model and isolated langendorf-perfused rat heart model [77]. RNase1 is ubiquitously expressed and the presence of highly specific RNase inhibitors limits its cytotoxicity in the cells. Apart from the gastrointestinal tract, which is the primary site for RNase1 production, vascular endothelial cells also produce RNase1 [78] primarily during stress [79]. Previously Cabrera-Fuentese and colleagues demonstrated that, RIPC prior to cardiac surgery decreased pathological eRNA and TNF-α expression in patients which was otherwise more than 20-fold and 7-fold higher without RIPC, respectively [80]. Furthermore, RIPC increased the plasma RNase1 activity by more than 5-fold when compared with the control. It has been subsequently proposed that RIPC dependent endothelial release of RNase1 degrades eRNA and prevents its interaction with TNF-α and other proteins to limit the pathological features of I/R. However further studies that alter RNase1 activity in the RIPC model are required to further understand the molecular process of protection mediated by RNase1.

### Apolipoprotein A-I

Apolipoprotein A-I (ApoA-I) is the major component of high-density lipoprotein in plasma. Hilbert *et al*. previously demonstrated the potential for ApoA-I as a circulatory mediator of RIPC-induced cardioprotection in Wistar rats [81]. They found that, 10 min of limb reperfusion after 10 min of limb ischemia increased plasma ApoA-I by 30% compared to untreated control group, whilst 5 min of limb reperfusion after 10 min of limb ischemia did not change the plasma ApoA-I level compared to the untreated rats. When Apo-AI was injected as an intravenous bolus (10mg/kg) 10 min prior to ischemia for 40 min and a reduction of almost 15% in MI size was reported after 2 h of reperfusion. A rat plasma proteomic study by Lang *et al.* did not report differential expression of ApoA-I in blood plasma after RIPC [32]. However, there were key differences between the two studies; Hibert *et al.* used renal artery occlusion whereas Lang *et al.* used hind limb occlusion to induce RIPC effect. In addition, the reperfusion time after ischemia was also different between the two studies. The beneficial effect of ApoA-I may be attributed to its ability to scavenge the TNF-α released by the myocardial tissue. Also, intravenous administration of ApoA-I prior to renal pedicle ischemia and prior to myocardial reperfusion limits the I/R-mediated injury. This includes reduced cytokine production by the injured tissue and suppressed endothelial ICAM-1 expression along neutrophil adherence and migration. Though the molecular mechanisms behind the increased expression of ApoA-I in the blood remains unclear, the quick response to RIPC by increasing the plasma concentration of ApoA-I led to a theory that suggests that RIPC does not increase the level of new ApoA-I synthesis but mobilizes the existing ApoA-I. Further studies are required to delineate the proposed theory.

### MicroRNAs

MicroRNAs (miRNA) are a class of endogenous small non-coding RNAs that circulate in a stable form in blood and regulate posttranslational gene expression. Previously, Li and colleagues reported that circulating microRNA-144 (miR-144) plays an important role in MI sparing effect of RIPC in mice [82]. Previously Zhang *et al*. observed that overexpression of miR-144 and miR-151 augmented cytoprotection in response to simulated I/R injury [83]. Also, local IPC-mediated myocardial functional recovery was lost in antagomiR-51 treated hearts in a murine model but failed to mimic similar result in miR-144 treated hearts suggesting that miR-144 may not be an important regulator of cytoprotection in IPC. Li and colleagues followed up by exploring the role of miR-144 in RIPC. They demonstrated that RIPC increased plasma miR-144 in both mice and human. In addition, RIPC also showed an increase in myocardial expression of miR-144 in mice, whereas I/R injury decreased myocardial expression of miR-144. Intravenous administration of miR-144 conferred both early (I/R injury immediately after miR-144 administration) and delayed (I/R injury after 3 consecutive days of miR-144 administration) cardioprotection whereas antisense oligonucleotide targeting miR-144 (antagomiR-144) abolished the RIPC effect on the heart. Microvesicles and exosomes are the major carrier of circulating miRNA, however analysis of the active constituent of the micro-particles (50-400 nm) showed no significant increase in micro-particle numbers and miR-144 which contradicts with the finding of Giricz *et al*. [72]. This may be due to differences in microparticle isolation, purification, microparticle size and different protocols of RIPC. Conversely, the exosome pellet showed almost 4-fold increase in miR-144 precursor and an increase in miR-144 in exosome-poor serum [72]. This group then demonstrated that miR-144 binds with Argonaute-2, a known extracellular miRNA carrier. Although the source of miR-144 and the exact mechanism of action of miR-144 in the heart are not clear, the phosphorylation of protective kinases such as Akt, GSK-3β, p42/44 MAPK increased while inducing autophagy signaling as early as 1 h after administration of miR-144. These findings suggest a vital role of miR-144 as a circulating mediator of RIPC and plasma miR-144 may be a potential biomarker for the successful application of preconditioning, aiding the study of efficacy of RIPC and abrogation of RIPC in clinical trials.

Mingua and colleagues studied nine microRNAs including miR-150, miR-21, miR-195, miR-132, miR-140, miR-144, miR-24, miR-214, miR-34a and found that RIPC significantly upregulated only miR-24 in the rat plasma exosomes [84]. Moreover, miR-24 down-regulated pro-apoptotic Bim protein expression and conferred cardioprotection. However, this group did not observe any alteration in miR-144 level in the exosomes after RIPC. Though the RIPC stimulus protocol in this study was the same as Li and colleagues [82], the disparity in miR-144 level after RIPC may be attributable to species difference.

### Nitrite

Ischemia and shear stress depolarizes cell membrane and inhibits the function of K^+^ channels. Inhibition of K^+^ channels lead to activation of Ca^2+^ channels and increased Ca^2+^ influx into endothelial cells and activates Ca^2+^ dependent endothelial nitric oxide synthase (eNOS). Pharmacological and genetic techniques revealed that, eNOS in the endothelial cells are the source of nitrite production during reactive hyperemia, which is then readily transported to the target organ especially to the myocardium. RIPC does not phosphorylate myocardial Akt and Erk proteins, which supports the notion that nitrite in the heart is remotely produced [85]. Hypoxia and ischemia inhibits oxidative phosphorylation, impairs ATP production and activates xanthine oxidase [86]. Davidson and colleagues reported that glyceryl trinitrite (GTN) patch (0.026 mg/h) reduced MI size in mouse model and improved blood pressure [87]. The degree of protection achieved by GTN patch was similar to that achieved by 3 cycles of 5 min RIPC. Similarly, a recent study by Hauerslev *et al*., suggests that long term application of GTN or RIPC is protective against ischemia-reperfusion injury, however a combined treatment eliminates this protective effect, suggesting a clinically relevant interaction [88]. Previous findings in humans, reported that forearm intermittent I/R modulates plasma NO^·^ / nitrite levels [89, 90]. Nitrite, the oxidation product of NO^·^ is a stable reservoir of NO^·^ and has a half-life of approximately 35 min [91]. Rassaf and colleagues demonstrated that, reactive hyperemia is essential to significantly improve the eNOS derived plasma nitrite levels. In addition, eNOS^-/-^ mice totally abrogated the MI sparing effect as well as diminishing the RIPC linked increase of plasma and myocardial nitrite levels. They also matched RIPC-mediated increased level of circulating nitrite with exogenous supplementation of intravenous nitrite and reported similar level of cardioprotection as RIPC [85]. These findings shed further light on why the effect of RIPC is not as pronounced in humans with pathological conditions such as atherosclerosis and coronary artery disease, given that endothelial function in these patients is partly impaired and impacts circulating nitrite levels.

### Calcitonin Gene-Related Peptide (CGRP)

Recent studies have characterized the involvement of capsaicin sensitive sensory neurons in RIPC [92, 93]. Sensory neurons are widely spread throughout the mammalian cardiovascular system and contain cardio-stimulatory neuropeptides. Among the neuropeptides, calcitonin gene-related peptide (CGRP), substance P and other neurokines play an intricate role in modulating cardiac function through local cardiac reflexes [94–96]. CGRP release is regulated by multiple factors such as brief ischemia, hyperthermia or autocoids [97–99]. Plasma levels of CGRP are increased by brief anterior mesenteric artery occlusion [100]. Furthermore, CGRP is involved in facilitating preconditioning-induced protection [101–103]. Similarly, it confers early and delayed cardioprotection against reperfusion injury *in vivo* [100] and in isolated rat heart [101]. CGRP facilitated early preconditioning is thought to involve only the release of CGRP whilst delayed preconditioning involves increased α-CGRP but not β-CGRP expression via NO and/or CO pathways [104]. Wolfrum and colleagues explored the role of CGRP in MI *in vivo* and delineated its role in the transduction mechanism of RIPC to the heart [105]. This group remotely preconditioned rat heart by occluding the mesentery artery for 15 min followed by 15 min reperfusion. RIPC by mesentery artery occlusion (MAO) in rats reduced MI size by around 40% and increased plasma CGRP levels by almost 40%, which was comparable to the plasma level of CGRP after pharmacological preconditioning by low dose of CGRP. Even though CGRP has a vasodilatory effect, a low dose of CGRP is protective against MI and limits infarction size without affecting hemodynamics. In addition, ganglion blocker hexamethonium did not prevent CGRP release after mesenteric preconditioning, but it abolished CGRP-mediated activation of myocardial PKCε and proved that neural response to RIPC is due to the systemic release of CGRP [105]. Peng and colleagues used CGRP receptor antagonist, CGRP_8-37_, to show that ischemic preconditioning-mediated cardioprotection involves CGRP whilst pre-treatment with CGRP receptor antagonist CGRP_8-37_ reversed the MI size limiting effect and PKCε translocation [106]. These findings suggest that CGRP is a humoral mediator of RIPC which further targets downstream neural pathways to confer cardiac protection.

### Interleukin-10

RIPC has been shown to modulate gene expression for proteins involved in inflammatory responses such as leukocyte chemotaxis, adhesion to endothelial cells, migration, exocytosis, cytokine synthesis, innate immune response and apoptosis in circulating human neutrophils [41, 107]. Late RIPC inhibits inflammatory responses in reperfused tissues. Interleukin-10 (IL-10) is an anti-inflammatory cytokine that decreases production of chemokines and cytokines [108] and can activate pro-survival pathways [109, 110]. IL-10 has two receptor subunits IL-10R1 and IL-10R2. IL-10R1 is expressed by all IL-10 responsive cells whereas IL-10R2 is ubiquitously expressed. IL-10R1 receptors are expressed on cardiomyocytes [111]. Late RIPC reduced infarct size and increased left ventricular developed pressure (LVDP), which was reversed by treating the heart with IL-10 receptor antibodies [111]. RIPC did not have cardioprotective effects on IL-10 knockout mice and on wild type mice treated with IL-10 receptor antibodies blocking the binding of IL-10 to IL-10R1. However pretreatment of IL-10 knockout mice with recombinant IL-10 significantly decreased infarct size [111]. Furthermore, the size of IL-10 (18 kDa) is consistent with notion that humoral mediators are likely to be <30 kDa [108]. Increased plasma IL-10 levels can quickly accumulate in tissue due to IL-1 receptor expression and high affinity for IL-10 on cardiomyocytes. Conversely, IL-10 can increase its own expression in tissue [112, 113]. Cai and colleagues showed that late RIPC increased plasma and cardiac IL-10 level at 24 h and activated Akt-eNOS signaling pathway through IL-10 receptors in myocardium while upregulating IL-10 expression in preconditioned skeletal muscle [111]. Whether increased plasma and cardiac IL-10 is released from the preconditioned skeletal muscle remains unclear.

### Interleukin-1 Alpha

Interleukin-1 Alpha (IL-1α) is an inflammatory mediator and released by macrophages, monocytes, endothelial and epithelial cells [114, 115]. Lugrin and colleagues demonstrated in mice MI model that, cardiomyocytes also release IL-1α into the systemic circulation without elevating the myocardial IL-1α level [116]. IL-1α blocking and IL-1α receptor antagonist has been reported to reduce myocardial I/R injury [117–119]. Gedik and colleagues reported that, RIPC on the upper arm of human significantly increased circulating IL-1α compared to placebo and remained increased until further cardioplegic arrest [120]. As both CABG surgery and RIPC have a systemic response [121–123], the study by Gedik and colleagues [120] raised the question whether the observed increase in IL-1α is due to I/R injury or RIPC. It has been suggested that, IL-1α may indirectly activate cardioprotective signaling pathways by IL-6 induction [124]. Preconditioning and pretreatment with exogenous IL-1α in isolated perfused rat heart improved myocardial function and reduced MI size suggesting the cardioprotective effect of circulating IL-1α [125–127].

### Stromal cell derived factor-1α

Stromal cell derived factor-1α (SDF-1α) is a 10 kDa CXC chemokine involved in recruiting bone marrow derived stem cells at the site of myocardial injury [128]. SDF-1α can bind to its receptor CXCR4 and protect the isolated heart from acute injury [129]. SDF-1α can also protect the heart from acute injury without engaging the stem cells, either through direct intracardiac administration prior to ischemia *in vivo* or after perfusion prior to ischemia in isolated hearts *ex vivo*. SDF-1α levels increase in response to hypoxia [130]. RIPC has been reported to increase plasma SDF-1α levels by 50% in rats and SDF-1α treatment confers cardioprotection in isolated papillary muscles. This protective effect was abolished by AMD3100, a highly specific inhibitor of CXCR4. In addition, AMD3100 prevented the induction of RIPC-induced cardioprotection in isolated perfused hearts suggesting the involvement of SDF-1α in RIPC [131]. However, proteomic analysis of human plasma samples confirmed that SDF-1α was not upregulated after RIPC stimulus [132]. SDF-1α has a plasma half-life of about 25 min [133]. However, remote postconditioning increased SDF-1α by 1 h, returning to basal level by 3 h in female Sprague-Dawley rats [134]. Conversely, similar procedure was reported to increase SDF-1α by 1 h and 3 h in mice [135]. It is possible that preconditioning-mediated SDF-1α regulation and expression are species and time specific. The SDF-1α-/CXCR4 axis is involved in the activation of JAK-STAT, PI3K, and MAPK pathways which are well known mediators of RIPC-induced cardioprotection [129, 136, 137]. Nonetheless, it is not clear whether the endogenous increase in SDF-1α is sufficient to stimulate RIPC effect *in vivo*. Further experiments are essential to explain whether SDF-1α is an RIPC mimetic or can only be used as a biomarker for RIPC optimization.

### Glucagon-like peptide 1

Glucagon-like peptide 1 (GLP-1) is a human incretin hormone, released by the intestine in response to food ingestion [138, 139]. GLP-1 release in regulated by vagal efferent activity [140, 141]. GLP-1 activity is mediated by GLP-1 receptor (GLP-1R) dependent or independent manner [142, 143]. Several studies have reported the cardioprotective effect of GLP-1 in animal model [142, 144–146]. GLP-1 therapy in clinical settings has also proven to be successful in limiting the reperfusion injury [147, 148]. The efficiency of GLP-1R agonists in mediating cardioprotection has also been demonstrated in human [149]. However, the role of GLP-1R in the activation of cardioprotective signaling pathways remains controversial. Basalay and colleagues demonstrated that RIPC conferred cardioprotection by GLP-1R activation and involves M3 muscarinic receptor dependent mechanism [150]. RIPC increased plasma GLP-1 level [150] however, whether this increase in plasma GLP-1 is physiological relevant warrants further discussion. GLP-1 has a low half-life (couple of minutes) and majority of the intestine-produced GLP-1 is rapidly degraded [151, 152]. However, GLP-1 has a longer lasting beneficial effect even when the circulating level drops to normal level [151]. Previously, Pyke and colleagues suggested that ventricular cardiomyocytes do not express GLP-1R [153]. Nonetheless, GLP-1 and GLP-1R agonists have been reported to have actions in cells and tissues that do not express GLP-1R [154, 155]. In addition, numerous studies have described the direct action of GLP-1R agonists on the myocardium [155]. It is possible GLP-1 mediates RISK pathway activation in the cardiomyocytes via GLP-1 binding to remote GLP-1R on endothelial or smooth muscles cells triggering a paracrine response [139], releasing cardioprotective factors that act on the cardiomyocytes. There is controversy whether the protective effect of GLP-1 therapy can be replicated with RIPC. GLP-1 protects against ischemic left ventricular dysfunction and myocardial stunning following coronary balloon occlusion during elective PCI [156, 157], however RIPC does not demonstrate similar effect [158].

### Amino acids

Multiple studies have implicated amino acids as the circulating mediator of RIPC. Alpha-ketoglutarate-dependent dioxygenase Egln1 is ubiquitously expressed and is the primary regulator of HIF-1α [159]. Egln1 acts as an oxygen sensor and ischemia inactivates Egln1 [159]. Olenchock and colleagues have demonstrated that Egln1 inhibition in skeletal muscle conferred cardioprotection from I/R injury [160]. This group reported that acute inactivation of Egln1 activity leads to rapid induction of alpha ketoglutarate and systemic level of alpha ketoglutarate increases with declined activity of Egln1. Alpha ketoglutarate is metabolized to kynurenic acid by the liver [160]. Frezza and colleagues demonstrated that, kynurenic acid precursor, kynurenine is upregulated in hypoxic cells [161]. Chao and colleagues reported that, hind limb and forearm preconditioning increase circulating kynurenic acid in rats and human respectively [162]. Exogenous kynurenic acid limits the MI size [160]. However, kynurenic acid level has been found to be high after cardiac arrest in both animal model and in humans [163], underscoring a potential role of kynurenic acid metabolism in RIPC setting.

Glycine, the smallest of the amino acids, protects from I/R-induced necrotic and apoptotic cell death in different target organs and cell types including kidney [164, 165], lung [166], cardiomyocytes [167] and hepatocytes [168]. Glycine has been demonstrated to delay I/R-associated sarcolemma rupture in hepatocytes [169]. Furthermore, glycine prevented mPTP opening in rat isolated mitochondria after I/R [167]. Moreover, glycine prevented myocardial necrosis after prolonged hypoxia or hypoxia-reoxygenation in isolated rat heart [167, 170]. It has been demonstrated that, RIPC increased circulating glycine level in the blood plasma of a pig model and glycine receptor antagonist pretreatment abolished the protective effect of RIPC plasma dialysate in isolated mice heart, whereas glycine pretreatment reduced MI size [171]. Chao and colleagues reported that, RIPC increased glycine in rat and human plasma, and intraperitoneal delivery of glycine prior to myocardial ischemia reduced MI size in rat [162]. It is possible that glycine receptor activation is involved in RIPC-mediated cardioprotection. However, lower concentrations of glycine failed to confer cardioprotection suggesting that glycine may be essential but not the only mediator of RIPC.

## CLINICAL TRIALS

Multiple studies are currently on-going to assess the efficacy of RIPC in protecting the heart on multiple clinical outcomes. Su and colleagues have completed a trial on 86 participants to assess the effect of RIPC in patients undergoing CABG, with a primary aim of measuring serum troponin T level over 72 h post CABG (NCT03010839) and yet to report the findings. Wang and colleagues have completed a randomised trial with 66 participants to verify the myocardial protective effect of RIPC in patients undergoing off-pump coronary artery and exclusion criteria includes LVEF < 35, AMI and multiple organ dysfunction (NCT03340181) but yet to report. The primary endpoint of this study is the ICU staying time of the patients after surgery. Similarly, Thielmann and colleagues are recruiting an estimated 800 participants for a phase 2 and phase 3 Germany-based randomised trial to study the effect of RIPC in patients undergoing on-pump CABG with non-propofol anaesthesia and crystalloid cardioplegic arrest with the primary outcome of assessing perioperative extent of myocardial injury by measuring serum troponin I level over 72 h post CABG surgery (NCT01406678). In addition, Voisine and colleagues are recruiting 140 participants for a randomised trial with a primary objective to verify if RIPC prior to aortic valve replacement surgery can confer cardioprotection. This study aims to measure the change in troponin T-HS and CK-MB concentration at 6, 12, 24 and 48 h post-operatively (NCT03305094). Yellon and colleagues are recruiting 200 participants to study the effect of RIPC in type II diabetic patients undergoing CABG (NCT00397163). An important facet of this trial is the focus on understanding the RIPC-effect on diabetic CABG patients. PREP (Pediatric remote ischemic pre-conditioning prior to complex cardiac surgery), a randomised pilot study, recruited 53 participants and aims to assess if RIPC prior to heart surgery can improve the recovery of heart and brain after heart surgery in newborn babies with congenital heart disease (NCT01739088). This study aims to assess the feasibility for a larger randomized controlled study on paediatric RIPC. Deja and colleagues have completed a phase 2 trial on 134 participants who were presented with stable coronary artery disease referred for CABG (NCT01994707). The findings of the trial is yet to be reported. The study aimed to assess if RIPC changes the extent of apoptosis in human right atrial appendage and myocardial biopsy samples from LV harvested during cannulation for CABG. This study was performed under controlled experimental setting with the aim to establish if the RIPC phenomenon can be effectively used in clinical practice. RICARDO (Remote Ischemic Conditioning to Attenuate Myocardial Death and Improve Operative Outcome), a phase 2 clinical trial is currently recruiting an estimated 80 participants (NCT03363958). The interventional group will recipe RIPC 24 h prior to off-pump CABG and immediately prior to CABG. The RIPost group will receive intermittent lower limb ischemic conditioning within 60 min after the surgery, whereas control group will receive sham procedure perioperatively. The primary outcome measures of this study are postoperative myocardial necrosis at 72 h postoperatively and postoperative kidney injury at 7 days postoperatively.

Two large randomized controlled trials- CONDI-2 (Effect of RIPC on Clinical Outcomes in STEMI Patients Undergoing pPCI; NCT01857414) and ERIC-PPCI (Effect of Remote Ischemic Conditioning on clinical outcomes in STEMI patients undergoing PPCI; NCT02342522) are recruiting an estimated 2600 and 2000 participants respectively, randomized to RIPC or sham are nearing completion with harmonized primary endpoint of cardiac death and hospitalization for heart failure at 1 year in STEMI patients treated with PCI. ERIC-PPCI clinical trial includes two sub-studies: the biomarker sub-study will investigate enzymatic infarct size at 6 months and the cardiac magnetic resonance (CMR) sub-study will investigate infarct size at 6 months. RECOND (Reduction in Infarct Size by Remote Per-postconditioning in Patients With ST-Elevation Myocardial Infarction), a randomized trial assessing the effect of RIPC prior to PCI has completed study with 120 participants and will report on the changes in myocardial infarct size at 1 week determined by CMR (NCT02021760). RIC-STEMI (Remote ischemic conditioning in ST-elevated Myocardial Infarction as Adjuvant to Primary Angioplasty) has recruited 516 patients and aims to assess the effect of remote ischemic conditioning on cardiac-related death or hospitalization for heart failure rates up to 1 year during PCI in patients for STEMI (NCT02313961). CORIC-MI (Comprehensive Remote Ischemic Conditioning in Myocardial Infarction) is recruiting an estimated 200 participants to evaluate whether comprehensive remote ischemic perconditioning (5 cycles of 5 min inflation and 5 min deflation of cut on a lower limb prior to PCI), postconditioning (5 cycles of 5 min inflation and 5 min deflation of cuff on a lower limb immediately after PCI) and delayed ischemic conditioning (5 cycles of 5 min inflation and 5 min deflation of cuff on a lower limb daily on 2-28 days post MI) as an adjunctive therapy in STEMI patients undergoing PCI can improve LVEF and remodeling at 30 days assessed by CMR with a minimum follow up time period of 1 year [172](NCT03233919). DREAM (Daily Remote conditioning in Acute Myocardial Infarction), a UK based multicenter randomized controlled phase 2 trial has completed the study on estimated 90 participants (NCT01664611). This study focused on evaluating the effect of daily RIPC for 4 weeks post MI. The inclusion criteria included previous STEMI, successful PCI and LVEF<45% on baseline echocardiography. The primary outcome data are obtained from baseline and 4 month CMR to assess the changes in LVEF. CRIC-RCT (Chronic Remote Ischemic Conditioning to Modify Post-MI Remodeling) is a multicenter clinical trial is currently recruiting an estimated 20 participants who are presented with STEMI involving LAD within 12 h of onset of the symptoms (NCT01817114). In this study, the effect of 4 cycles of 5 min-each ischemia and reperfusion of the right arm prior to PCI will be evaluated by assessing the changes in left ventricular end diastolic volume (LVEDV) at 28 days post PCI by MRI. This clinical trial excludes diabetic patients.

CONDI-PET (Effects of Remote Ischemic Conditioning on Myocardial Perfusion in Humans), a non-randomized phase 3 clinical trial in Denmark has completed the study on 50 participants with the primary objective of assessing the feasibility of using RIPC to treat ischemic heart disease and soon to report on changes in myocardial blood flow following intervention in these participants (NCT02230098). ERIC-LYSIS (Effect of Remote Ischemic Conditioning in Heart Attack Patients) has completed a randomized controlled trial on 519 participants to study whether remote ischemic conditioning in patients with STEMI can reduce myocardial tissue damage thereby preventing the onset of heart failure. The primary aim of this study was to measure serum CK-MB and troponin T levels at different time points post thrombolysis to estimate the extent of myocardial damage during a heart attack (NCT02197117).

A phase 2 clinical trial is underway, studying the protective effect of RIPC on heart, kidney and brain in patients undergoing transcatheter aortic valve implantation (TAVI) on 100 recruited participants (NCT02080299). The primary endpoint of this study is peri-interventional myocardial injury by assessing the changes in troponin I serum concentrations at 72 h post intervention. We speculate that once these randomized trials are completed and the findings are reported, we will be in a better position to address the role of RIPC in protecting the heart from reperfusion injuries, and remodeling and whether this non-invasive technique merits phase 3 randomized trials before widely using in patient care.

## CHALLENGES OF TRANSLATING RIPC IN CLINICAL SETTINGS

RIPC as a clinical strategy have been widely investigated in a large number of clinical trials with varied results. A systematic review and meta-analysis by Mcleod and colleagues suggested RIPC as a promising adjunctive treatment to PCI for the prevention of STEMI-related reperfusion injury [173]. Table 1 includes some clinical trials on RIPC that did not render the expected cardioprotective outcome in CABG and PCI. Loss of translation in RIPC is a multi-faceted problem. Many of the trials were small cohort size, single-centered, had short follow-up period and lacked the power to investigate the end points such as death or hospitalization.

We have discussed the role of different proteins as circulating mediators of RIPC-mediated cardioprotection, however proteins in general have several significant therapeutic limitations. Protein therapeutics are expensive due to its high production cost limiting its patient access and clinical applications [174]. Chemical and physical instability of the therapeutic proteins is a critical issue and require further attention. Proteins can undergo degradation via various processes such as aggregation, denaturation, hydrolysis, oxidation and racemization. The route of administration of therapeutic proteins is also crucial to translate these humoral mediators of RIPC in clinical practice. Whilst intravenous, subcutaneous and intramuscular routes bypass the gastrointestinal enzymatic degradation, subcutaneous and intramuscular routes significantly reduces the bioavailability of these proteins compared to other routes [175]. A major concern for recombinant proteins as a therapeutic strategy is immune response during an unpredictable time scale which may reduce the efficacy of the therapeutic protein or rarely inactivate the native endogenous proteins [176]. Though this review paper has discussed the preclinical potential of the humoral proteins in reducing cardiac injuries from I/R injury, the mechanistic understanding and the pharmacodynamic and pharmacokinetic information of these potential candidates is very limited to translate it to clinical practice.

In addition, comorbidities such as hypercho-lesterolemia, diabetes, hypertension, left ventricular hypertrophy, obesity, age, sex, chronic kidney disease, comedication can limit the effectiveness of RIPC in clinical settings [177]. Hypercholesterolemia was the first comorbidity reported to impair the preconditioning effect. Tang and colleagues reported that, hypercholesterolemia impairs the upregulation of tetrahydrobiopterin (BH4) and reduces the nitric oxide synthase level [178]. Reduced bioavailable NO and increased peroxynitrite has been reported to reduce RIPC-induced cardioprotective effect through MMP-2 upregulation [179].

Diabetes macrovascular complications is associated with cardiovascular diseases. Yetgin and colleagues provided an indirect evidence for the failure of ischemic post-conditioning in diabetic patients undergoing PCI for AMI, where multiple balloon inflations as post-conditioning stimulus failed to induce cardioprotective effect [180]. Jensen and colleagues found that, RIPC in non-diabetic healthy individuals, but not diabetic patients with neuropathy, release some undefined humoral factors and this humoral factor can increase myocardial ischemia tolerance in rabbits [181]. May be it is associated with the impaired cardioprotective pathways in diabetes such as PI3K-Akt, ERK1/2, K ATP dysfunction and reduced CGRP release [182].

Majority of the AMI patients present with hypertension [183]. Prodromal angina [184] and pre-infarct angina [185] in hypertension patients did not show any protective effect of preconditioning. However, pre-infarction angina is a common feature in STEMI patients and it may confer cardioprotection [186]. It is possible that preexisting cardioprotection by pre-infarction angina limit the potential for further cardioprotection by RIPC.

Myocardium undergoes significant structural and functional changes with age. Previous studies reported significant reduction in ischemic tolerance [187] and prognosis following AMI [188]. A retrospective study reported that, pre-infarct angina was reduced in elderly patients [189]. Heinen and colleagues reported that blood-borne mediator of RIPC in human is affected by both age and sex [190]. They demonstrated that, RIPC plasma of male volunteers reduced infarct size in both young and old rats whereas RIPC plasma from old male volunteers failed to reduce infarct size in male rats. In contrast, RIPC plasma from both young female and old female volunteers was unable to reduce infarct size in male rats compared to control female blood plasma. As estrogen can produce cardioprotective effect [191], it is possible that estrogen receptor activation masks the cardioprotective effect of RIPC plasma.

Chronic kidney disease (CKD) is a common comorbidity in cardiovascular diseases [192] and is associated with impaired myocardial ischemic tolerance [193] [194]. However, a meta-analysis by Zhou and colleagues suggested that, RIPC reduced acute kidney injury in adults undergoing cardiac surgery [195]. Er and colleagues confirmed in RenPro Trial that RIPC stimulus also reduced contrast medium-induced acute kidney injury in high risk patients [196]. However, a meta-analysis by Xie and colleagues suggested that, whilst RIPC reduced post cardiac surgery troponin I and troponin T release, it could not reduce the incidence of AMI, AKI, and mortality [197]. However, this particular meta-analysis did not consider few confounding factors including whether the patients were on or off pump during the CABG, comorbidity such as diabetes or hyperlipidemia and use of specific medications. Chronic kidney disease does not seem likely to significantly impair the RIPC effect, but a larger multi-center trial is needed to confirm the findings.

Patients undergoing concurrent valve surgery with CABG experience larger surgical trauma compared to CABG surgery alone and are less likely to get the full benefit of RIPC [198]. RIPC mostly protect the heart from acute I/R injury, however, cardiac surgery-related injuries are not limited to I/R injury but are multi-factorial including inflammation, direct handling of the heart and coronary micro-embolism [198]. Hence, the efficacy of RIPC is linked with the complexity of the surgery.

The extent of preconditioning stimulus also modulates the cardioprotective effect of RIPC. Verouhis and colleagues also reported a neutral effect on MI size in anterior STEMI patients who underwent RIPC consisting of different numbers and cycles of leg ischemia and reperfusion before and after coronary reperfusion [199]. Too much of preconditioning stimulus may augment rather than attenuate the myocardial I/R injury [200].

Current guidelines recommend PCI to be performed within the first 12 h after the onset of the symptoms [201], and STEMI patients can obtain maximum benefit from myocardial reperfusion when done within the first 6 h after the onset of the symptoms [202]. However, whether RIPC follows the same time frame is unknown. Interestingly, area at risk (AAR) is critical to obtain the beneficial effect of RIPC. Infarct size is dependent on the AAR [203, 204] and current clinical evidence suggests that patients with larger AAR (greater than 40% of the LV volume) are more likely to obtain protection from RIPC [202]. Patients with small AAR develop smaller infarcts and are less likely to benefit from RIPC. Hence, it is crucial to accurately measure the infarct size and AAR. Imaging techniques such as cardiac MRI is emerging as a powerful imaging technique which is likely to address the challenges of measuring the accurate AAR and MI size and can be used in assessing alternate outcomes of cardioprotection [202].

Anesthesia is a confounder of cardioprotection by RIPC [205]. Interestingly, propofol anesthesia did not show significant differences in the primary outcome parameters including cardiovascular health, myocardial infarction, coronary revascularization, stroke and post-operative troponin I release in more than 90% of the CABG patients included in ERICCA trial [206]. However, perioperative anesthetic regimen was not defined in this study and in numerous cases, propofol was used in combination with opioids as the hypnotic agent. Another multicenter trial RIPHeart used propofol as the only narcotic agent while completely avoiding the use of any other volatile anesthetics [207]. Both ERICCA and RIPHeart trials used 4 cycles of 5 min arm occlusion and 5 min reperfusion rather than the established 3 cycles of 5 min intermittent occlusion and reperfusion and reported a neutral outcome in infarct size reduction in patients with anterior STEMI. Kottenberg and colleagues reported on a smaller single center trial demonstrating reduced postoperative Troponin I release only when anesthetic regimens without propofol was used [208]. A meta-analysis by Zangrillo and colleagues reported that volatile anesthesia, but not propofol, combined with RIPC reduced post-operative mortality rate in patients undergoing cardiac surgery [209]. It is still unclear how propofol anesthesia impairs the cardioprotective effect of RIPC but it is hypothesized that, propofol may be impairs the RIPC effect by abolishing the myocardial STAT5 phosphorylation [210] and/or by impairing the activation of the sensory fibers [211]. Interestingly, a multicenter trial in 240 patients undergoing cardiac surgery where thiopental was used for induction and sevoflurane for maintenance of general anesthesia reported that RIPC significantly reduced acute kidney injury (AKI) [123]. The results of this study have since been supported by a meta-analysis detecting statistically significant reduction in the incidence of post cardiac surgery AKI in patients receiving anesthetic regimen without propofol [212]. However, according to a meta-analysis by Zhou and colleagues alternative use of volatile anesthetics also attenuate the cardioprotection afforded by RIPC [213]. Propofol is the standard sedative agent used worldwide, hence completely avoiding propofol in the anesthetic regimen is limited to experimental settings but not to clinical settings which further complicates the translation of RIPC in clinical settings.

Co-medication can also act as a confounder of RIPC. CABG patients receive medications such as anti-diabetic medicines, statins, angiotensin-converting enzyme inhibitors, angiotensin receptor blockers, beta-blockers, morphine, calcium antagonists-which can interfere with the RIPC effect [205]. These medications may facilitate cardioprotection, which may obscure the potential of RIPC-mediated cardioprotection.

It is essential to conduct clinical trials with strict inclusion requirements. In order to increase the power, many of the clinical trials recruit high number of patients and follow up post-operatively for a shorter period of time. A large multi-center clinical trial is essential to investigate why some RIPC clinical trials failed to improve clinical outcomes in CABG [214], [215], [216] and PCI [217] patients.

## CONCLUSIONS

RIPC has provided an innovative non-invasive therapeutic strategy to prevent acute I/R injury in susceptible organs and tissues with some variability. Non-invasive procedures such as using a blood pressure measuring cuff around the arm to achieve protection against I/R injury has facilitated its translation from bench to bedside. Though there are several clinical trials that did not show beneficial effects of RIPC, further mechanistic studies will help us understand the underlying cause of the failure of these studies. Optimal modality, site and duration of RIPC remains unclear. RIPC may nonetheless benefit children and adults undergoing certain elective surgeries where there is potential to improve clinical outcomes. Future insights into the control of circulating mediators of RIPC, including transcriptional regulation and secretion into the bloodstream will assist the development of pharmacologic approaches stimulating protective signaling pathways in target organs.
